# Obesity and the onset of depressive symptoms among middle-aged and older adults in China: evidence from the CHARLS

**DOI:** 10.1186/s12889-018-5834-6

**Published:** 2018-07-24

**Authors:** Huiqiang Luo, Jijie Li, Qi Zhang, Peiya Cao, Xiaohui Ren, Aiping Fang, Haiying Liao, Lijuan Liu

**Affiliations:** 1 West China School of Public Health (No. 4 West China Teaching Hospital), Sichuan University, Chengdu, 610041 Sichuan China; 2 Department of Medical Records, West China Secondary University Hospital, Sichuan University, Chengdu, 610041 Sichuan China; 3 Department of Health Industry, Health and Family Planning Bureau of Wuhou District, Chengdu, 610041 Sichuan China; 4 Department of Health Related Social and Behavioral Science, West China School of Public Health (No. 4 West China Teaching Hospital), Sichuan University, No. 17 Section 3, Renmin South Road, Chengdu, 610041 Sichuan China

**Keywords:** Obesity, Depression, Chinese, Middle-age and older adults, CHARLS

## Abstract

**Background:**

The association between obesity and depression has been documented in previous systematic studies but remains controversial. Many prospective studies have focused on children and youth, and several studies have examined this relationship among older populations. This study of the changes in obesity status aimed to examine the association between depression and obesity among middle-aged and elderly adults in China.

**Methods:**

The data originated from the follow-up survey (2011 and 2013–2015) of the China Health and Retirement Longitudinal Study (CHARLS) and included 3337 residents aged at least 45 years who completed a physical examination and were evaluated with the Center for Epidemiological Studies Depression Scale (CES-D-10), which assessed depressive symptoms. Obesity status was defined by body mass index (BMI) and waist circumference (WC) according to Chinese criteria. A time-dependent Cox proportional hazards model was used to estimate the relationship between obesity status and depressive symptoms.

**Results:**

The rate of depression in men and women was 26.67 and 38.37%, respectively. Based on BMI, the proportion of the population that was overweight and obese was 28.07 and 9.26%, respectively, in males and 35.03 and 16.84%, respectively, in females. Males with obesity were less likely to suffer from depressive symptoms than males with a normal weight (ORHR = 0.506, 95% CI = 0.347~ 0.736). Based on WC, the proportion of abdominal obesity was 49.35% in males and 73.65% in females. Males with abdominal obesity were less likely to suffer from depressive symptoms than males without abdominal obesity (ORHR = 0.775, 95% CI = 0.644~ 0.933).

**Conclusion:**

Obesity is more likely to be associated with the onset of depression in males than in females. However, regardless of underweight or overweight status, the relationship between weight and depressive symptoms is negatively associated among females and males. In conclusion, both BMI and WC can be used as tools for examining the association between obesity and depression.

## Background

According to the World Health Organization, the prevalence of worldwide obesity has undergone a significant change since 1980 [1]. In the US, the prevalence of obesity accounts for one-third of the general population, and another one-third is overweight [2]. Similarly, the level and trend of obesity rates have also become a major public health concern in China [3]. Obesity is a major risk factor for heart disorders, hypertension, diabetes, and stroke [4]. Additionally, obesity is highly associated with decreased life expectancy, cognitive dysfunction, and reduced quality of life [5]. Some studies have reported that patients who are obese have increased need for healthcare utilization than normal-weight patients as a result of medication use and hospital care [6, 7].

Depression, which is recognized as a major chronic disease, has become a major health problem worldwide [8]. Depressive disorders make up a relatively significant share (4.5%) of the total disability-adjusted life years (DALYs) and account for 12.1% of the total years lived with a disability (YLDs) globally [9]. Depression is a common psychological disorder associated with high morbidity and is a serious economic burden for society in China [10].

The association between obesity and depression has been documented in previous systematic studies [11, 12] but remains controversial. Luppino et al. found that obesity at baseline could predict depression onset during follow-up based on a meta-analysis, and the results were most pronounced among Americans; meanwhile, another study reported no robust evidence of this association [13, 14]. Studies have found that both being underweight and obese have a significant impact on depression, whereas other studies have demonstrated that depressive symptoms are associated with being only underweight or obese [15, 16]. Moreover, the relationship between obesity and depression may differ by sex. Simon et al. found a stronger relationship between obesity and depression in females based on a cross-sectional study, but Nicole et al. reported that obesity, particularly visceral fat, increased the risk of onset of significant depressive symptoms only in males [11]. Additionally, prospective studies have examined the association between obesity and depression predominantly among children and youth, and a few studies have examined this relationship among older populations [12, 14].

In this study, the association between depression and obesity in both sexes was examined among middle-aged and elderly individuals in China using data from the China Health and Retirement Longitudinal Study (CHARLS). Our prior work using the 2013 cross-sectional data (i.e., CHARLS) indicated that the relationship between obesity and depression was negative in males and females [17]. The present study assessed whether the relationship between obesity and depression was still negative in males and females during the follow-up survey and whether changes in obesity had an impact on depression onset over time.

## Methods

### Data

The data used in this study came from the follow-up survey of the CHARLS targeting the middle-aged and older population (45+) in China. The CHARLS is an ongoing national longitudinal study administered by the National School for Development (China Center for Economic Research), with exams performed every 2 years for a total of 3 waves from 2011 to 2015. The first national baseline survey of the CHARLS was fielded between June 2011 and March 2012 and involved 17,705 respondents who were chosen randomly with a probability proportional to scale (PPS) in 450 villages/resident committees, 150 counties/districts and 28 provinces. The respondents were interviewed face-to-face in their homes via computer-assisted personal interviewing (CAPI) technology. Physical parameters, such as the respondents’ standing height, weight and waist circumference (WC), were measured by the trained investigators with standardized equipment (Index: height; Equipment: SecaTM213 Stadiometer Manufacturer/source: China Seca (Hangzhou) Co., Ltd. Index: weight; Equipment: OmronTMHN-286Scale Manufacturer/source: Krill Technology (Yangzhou) Co., Ltd. Index: waist circumference; Equipment: Soft Tape Measure Manufacturer/source: None.). The medical ethics committee approved the CHARLS study, and all interviewees were required to sign informed consent, Ethics approval for the data collection in CHARLS was obtained from the Biomedical Ethics Review Committee of Peking University (IRB00001052–11015). Ethics approval for the use of CHARLS data was obtained from the University of Newcastle Human Research Ethics Committee (H-2015-0290).

This study used the baseline data from 2011 and follow-up data from 2013 and 2015. To research the association between BMI (WC) and the onset of depression, we limited the samples to respondents who had no depression symptoms in 2011 and had received physical examinations in each of the three surveys. A total of 3337 subjects were included.

### Measurements

This study mainly focused on the association between obesity and depression. Previous studies have identified factors that not only had an impact on this relationship but also on obesity or depression alone, such as activities of daily living (ADLs), chronic disease, smoking and drinking, age, sex, education level, marital status, and Hukou status [1, 2, 13–17]. As a result, these factors were controlled for in this study.

### Depression

Depressive symptoms were assessed using the 10-item Center for Epidemiological Studies Depression Scale (CES-D-10) [18], which has been used to measure depressive symptoms among older adults and was validated among elderly respondents in China [19, 20]. The CES-D-10 contains 10 items with 4 response options: 1) rarely or none of the time (< 1 day); 2) some or few times (1–2 days); 3) occasionally or a moderate amount of times (3–4 days); and 4) most or all of the time (5–7 days). The values of the 4 options range from 0 to 3 successively. The total score ranges from 0 to 30, with a lower score indicating a lower level of depressive symptoms. A cutoff score ≥ 10 was used to identify the respondents who had significant depressive symptoms [17, 18].

### Obesity

Body mass index (BMI) and WC were used to define the obesity status of respondents.

BMI was used to describe general obesity. Respondents were categorized as underweight (< 18.5 kg/m^2^), normal weight (18.5 to 23.9 kg/m^2^), overweight (24.0 to 27.9 kg/m^2^), or obese (≥28.0 kg/m^2^) based on Chinese criteria [21].

WC was used to describe abdominal obesity. WC ≥ 85 cm in males and ≥ 80 cm in females was used to define abdominal obesity according to Chinese guidelines [21].

### ADL

ADLs were assessed with the ADL scale [22] using the following series of questions: “Because of a physical, mental, emotional or memory problem, do you have any difficulty with one type of everyday activity, excluding any that you expect to last less than three months?” The everyday activities included dressing, bathing or showering, eating, getting into or out of bed, using the toilet, and controlling urination and defecation. The response scale contained 4 options: 1) No, I don’t have any difficulty; 2) I have difficulty but can still do it; 3) Yes, I have difficulty and need help; and 4) I cannot do it. The participants classified as ADL independent had the ability to complete all of the activities without difficulty, whereas those who had difficulty on any item above were regarded as having an ADL disability.

### Chronic disease

Conditions of chronic disease were measured with the following question: “Have you been diagnosed with conditions listed below by a doctor?” The conditions included hypertension; dyslipidemia; diabetes or high blood sugar; cancer or malignant tumors; chronic lung diseases; liver disease; heart problems; stroke; kidney disease; stomach or other digestive disease; emotional, nervous, or psychiatric problems; memory-related disease; arthritis or rheumatism; and asthma. Each condition was assessed by trained investigators separately.

### Health behavior

The habit of smoking was measured with the question “Have you ever chewed tobacco, smoked a pipe, smoked self-rolled tobacco, or smoked cigarettes/cigars”, and the possible answers included the following 3 options: 1) Yes; 2) No; or 3) Quit. The habit of drinking was measured with the question “Did you drink any alcoholic beverages, such as beer, wine, or liquor in the past year, and if so, how often?”, and the possible answers included the following: 1) Drink more than once a month; 2) Drink but less than once a month; or 3) Do not drink.

### Sociodemographic characteristics

In this study, the sociodemographic characteristics included age, sex, education level, marital status, and Hukou status. Age was divided into two groups including 45 to 59 and 60 or older. Sex was categorized as male or female. Education level was categorized as illiterate, primary school, or junior high school and above. Marital status was categorized as married, cohabitating and divorced, separated, widowed, or never married. Hukou status (Hukou is a centuries-old Chinese word referring to the national household registration that Chinese governments have historically used to try to fix the population in place geographically) was categorized as rural or urban.

### Statistics analysis

Descriptive statistics on individual baseline characteristics were stratified by sex. A chi-squared test was used to compare dichotomous or categorical variables. The relationship between obesity and onset of depression was conducted by survival analysis. The endpoint was defined as the onset of depressive symptoms. In our study, no subjects were lost to follow-up or dropped out, so the subjects with no failure (depression) were censored at the end of the study. Time-dependent Cox proportional hazards models were used to estimate the unadjusted and adjusted hazard ratios (HRs) for the onset of clinically relevant depressive symptoms in relation to time-varying body weight status. Exhibiting depressive symptoms (1 = depressive symptoms, 0 = no depressive symptoms) was a dependent variable. Obesity status (BMI or WC) was a time-dependent independent variable. Age, education level, marital status, Hukou status, chronic disease, ADL, smoking and drinking were also included as control variables in the Cox models. The level of statistical significance was set at 95% (*P* < 0.05). Associations are presented as the HR with its 95% confidence interval (CI). Cox models were performed on the total study sample stratified by sex. Statistical analyses were conducted using SAS 9.3.

The general form of the Cox proportional hazards model was:

$$ h\left(t,X\right)={h}_0(t)\mathit{\exp}\left({\beta}_1{x}_1+{\beta}_2{x}_2+\dots +{\beta}_m{x}_m\right) $$

If we introduced intrinsic time-dependent covariates, the general model was converted to: *h*(*t*, *X*) = *h*_0_(*t*) *exp* (*β*_1_*x*_1_ + *β*_2_*x*_2_ + ⋯ + *β*_*m*_*x*_*m*_(*t*)).

where *x*_*m*_ is an intrinsic time-dependent covariate whose value changes with time, and *x*_*m*_ (t) represents the value of *x*_*m*_ at time t. *β*_*m*_ is the logarithm of the relative risk (HR) for each unit change in *x*_*m*_.

## Results

### Study population

The mean age of the participants was 57.78 (SD = 8.51) years, and 46.63% of the participants were female. Table 1 presents the baseline characteristics of the study cohort stratified by sex.

**Table 1:** Sociodemographic characteristics of the study cohort stratified by sex

Characteristics	Total	Female	Male	*P* value
	N (%)	N (%)	N (%)	
Total	3337	1556 (46.63)	1781 (53.37)	NA
Demographic				
Age (year)				
45~59	1985 (59.48)	1016 (65.30)	969 (54.41)	< 0.001
≥ 60	1352 (40.52)	540 (34.70)	812 (45.59)	
Marital status				
Married/cohabitating	3100 (92.90)	1419 (91.20)	1681 (94.39)	< 0.001
Divorced/separated/widowed/never married	237 (7.10)	137 (8.80)	100 (5.61)	
Socioeconomic status				
Education level				
Illiterate	637 (19.09)	467 (30.01)	170 (9.55)	< 0.001
Primary school and below	1452 (43.51)	645 (41.45)	807 (45.31)	
Junior high school or above	1248 (37.40)	444 (28.53)	804 (45.14)	
Hukou status (household registration)				
Rural	2699 (80.88)	1280 (82.26)	1419 (79.67)	0.058
Urban	638 (19.12)	276 (17.74)	362 (20.33)	
Health behavior				
Smoking				
Yes	1125 (33.71)	74 (4.76)	1051 (59.01)	< 0.001
No	1932 (57.90)	1463 (94.02)	469 (26.33)	
Quit	280 (8.39)	19 (1.22)	261 (14.65)	
Drinking				
Drink more than once a month	950 (28.47)	91 (5.85)	859 (48.23)	< 0.001
Drink but less than once a month	184 (5.51)	32 (2.06)	152 (8.53)	
Do not drink	2203 (66.02)	1433 (92.10)	770 (43.23)	
Health status				
Activity of daily living				
Independent	3100 (92.90)	1432 (92.03)	1666 (93.54)	0.091
Dependent	239 (7.10)	124 (7.97)	115 (6.46)	
Chronic disease				
0	1297 (38.87)	604 (38.82)	693 (38.91)	0.925
1	1023 (30.66)	473 (30.40)	550 (30.88)	
≥ 2	1017 (30.48)	479 (30.78)	538 (30.21)	
General obesity (BMI)				
Underweight	134 (4.02)	57 (3.66)	77 (4.32)	< 0.001
Normal	1731 (51.87)	692 (44.47)	1039 (58.34)	
Overweight	1045 (31.32)	545 (35.03)	500 (28.07)	
Obese	427 (12.80)	262 (16.84)	165 (9.26)	
Abdominal obesity (WC)				
Yes	2025 (60.68)	1146 (73.65)	879 (49.35)	< 0.001
No	1312 (39.32)	410 (26.35)	902 (50.65)	

The mean age of the enrolled females was 56.41 (SD = 8.46) years. The sample of females in this study was more likely to have a lower education level and to live with a spouse than the enrolled males. The mean age of the males enrolled in the study was 58.98 (SD = 8.38) years. The sample of males in this study was older and more likely to drink and smoke than the enrolled females.

In addition, a higher proportion of enrolled females were obese than males according to two different criteria. In terms of general obesity, the proportions of females who were overweight and obese were higher than those of males (overweight: 35.03% in females, 28.07% in males; obese: 16.84% in females, 9.26% in males). In terms of abdominal obesity, the proportion of obese females was higher than that of males (73.65% in females, 49.35% in males).

### Trend of obesity prevalence

Table 2 shows the changes in the prevalence of obesity at baseline and during both follow-up surveys.

**Table 2:** Distribution of body mass index and waist circumference categories by survey year and sex for CHARLS participants with no depressive symptoms at baseline

	2011	2013	2015
Female			
General obesity (BMI)			
Underweight	57 (3.66)	49 (3.15)	55 (3.53)
Normal	692 (44.47)	648 (41.65)	647 (41.58)
Overweight	545 (35.03)	574 (36.89)	591 (37.98)
Obese	262 (16.84)	285 (18.31)	263 (16.91)
Abdominal obesity (WC)			
Yes	1146 (73.65)	1264 (81.23)	1203 (77.31)
No	410 (26.35)	292 (18.77)	353 (22.69)
Male			
General obesity (BMI)			
Underweight	77 (4.32)	74 (4.15)	95 (5.33)
Normal	1039 (58.34)	984 (55.25)	960 (53.90)
Overweight	500 (28.07)	535 (30.04)	530 (29.76)
Obese	165 (9.27)	188 (10.56)	196 (11.01)
Abdominal obesity (WC)			
Yes	879 (49.35)	1011 (56.77)	949 (53.28)
No	902 (50.65)	770 (43.23)	832 (46.72)

In terms of general obesity, the proportion of overweight females monotonically increased from 35.03% in 2011 to 37.98% in 2015. However, compared with 28.07% in 2011, the proportion of overweight males underwent an instability fluctuation, declining to 29.76% in 2015 from 30.04% in 2013. The proportion of obese females also showed an instability fluctuation, increasing from 16.84% in 2011 to 18.31% in 2013 and then decreasing to 16.91% in 2015. However, the proportion of the male population with obesity monotonically increased from 9.27% in 2011 to 11.01% in 2015.

In terms of abdominal obesity, the proportion of the obese population among both males and females underwent an instability fluctuation. This proportion of obese females increased from 73.65% in 2011 to 81.23% in 2013 and declined to 77.31% in 2015, and the proportion of obese males increased from 49.35% in 2011 to 56.77% in 2013 and then decreased to 53.28% in 2015.

### Onset of depression

A total of 38.37% of the enrolled females who did not have depression initially experienced the occurrence of depressive symptoms during the follow-up survey; among males, this rate was 26.67%.

As Table 3 shows, the rates of depression onset decreased monotonically with obesity increases based on BMI, from 29.26% among normal-weight male participants to 24.00% among overweight male participants and 16.37% among male participants with obesity. The rate of depressive symptom onset in males with abdominal obesity based on the WC criteria was lower than that in males without abdominal obesity, from 29.49% among male participants with obesity to 23.78% among male participants without obesity.

**Table 3:** Association between obesity and depressive symptom onset by sex based on baseline obesity status

Characteristics	Female (N (%))		*P* value	Male (N (%))		*P* value
	No depressive symptoms	Depressive symptoms		No depressive symptoms	Depressive symptoms	
Total	959 (61.63)	597 (38.37)	NA	1306 (73.33)	475 (26.67)	NA
general obesity (BMI)						
Underweight	33 (57.89)	24 (42.11)	0.899	53 (68.83)	24 (31.17)	< 0.01
Normal	425 (61.41)	267 (38.59)		735 (70.74)	304 (29.26)	
Overweight	341 (62.56)	204 (37.44)		380 (76.00)	120 (24.00)	
Obese	160 (61.06)	102 (38.94)		138 (83.63)	27 (16.37)	
Abdominal obesity (WC)						
Yes	704 (61.43)	442 (38.57)	0.785	670 (76.22)	209 (23.78)	< 0.01
No	255 (62.19)	155 (37.81)		636 (70.51)	266 (29.49)	

Additionally, the rates of depressive symptom onset showed no significant association with obesity status in females, even though the association between obesity and depression in females showed a similar trend as that in males using two different criteria.

### Association between obesity and the onset of depression

Table 4 shows the results of time-dependent Cox proportional hazards models assessing the risk of depressive symptom onset during the follow-up survey according to the obesity status at baseline among participants without depression. Both crude and adjusted HRs for depressive symptom onset decreased as BMI and WC increased in males, but this result was not observed in females. After adjusting for demographic, socioeconomic, health behavioral and health status factors, males with obesity were less likely to suffer from depressive symptoms than normal-weight males according to BMI and WC (BMI: HR = 0.506, 95% CI = 0.347–0.736; WC: HR = 0.775, 95% CI = 0.644–0.933). However, there was no significant association between obesity status and depressive symptom onset according to BMI and WC among females.

**Table 4:** HR with 95% CI of depression according to the China-specific criteria of obesity based on BMI and WC stratified by sex^a^

	Female		Male	
	Crude HR (95% CI)	Adjusted HR (95% CI)	Crude HR (95% CI)	Adjusted HR (95% CI)^b^
Female				
general obesity (BMI)				
Normal	1.000	1.000	1.000	1.000
Underweight	1.099 (0.711~ 1.699)	1.089 (0.702~ 1.689)	1.048 (0.702~ 1.566)	1.058 (0.706~ 1.587)
Overweight	0.906 (0.755~ 1.087)	0.879 (0.731~ 1.057)	0.792 (0.643~ 0.974)*	0.858 (0.693~ 1.063)
Obese	0.982 (0.783~ 1.231)	0.906 (0.719~ 1.141)	0.514 (0.355~ 0.745)***	0.506 (0.347~ 0.736)***
-2Log likelihood	8508.942	8450.743	6953.708	6891.256
Abdominal obesity (WC)				
Yes	0.948 (0.778~ 1.154)	0.917 (0.752~ 1.119)	0.721 (0.602~ 0.863)**	0.775 (0.644~ 0.933)**
-2Log likelihood	8510.231	8452.458	6958.550	6899.773

Note. ^a^Tests for trend were conducted using time-dependent Cox proportional hazards models, treating BMI and WC as categorical and dichotomous variables, respectively

^b^Adjusted for age, marital status, education level, Hukou status (household registration), smoking, drinking, activities of daily living, and chronic disease

*P < 0.05; **P < 0.01; and ***P < 0.001

*HR* hazard ratio and *CI* confidence interval

## Discussion

In this study, we found that obesity decreased the onset of depression among middle- and older-aged males. However, there was no association between obesity and depression for middle- and older-aged females. This finding again revealed the existence of sex differences in obesity and depression Moreover, regardless of underweight or overweight status, we found that the relationship between weight and depressive symptoms was not significantly different among females and males. The finding of an association between obesity status and depression is consistent with previous epidemiological studies focusing on elderly people [23, 24]. Lawrence et al. examined the association between body weight and depression in 2245 males and females aged 50 to 89 years living in the US, and the results showed that depressive symptoms were inversely associated with body weight in males but not in females [24]. Crisp et al. concluded that there was a significant positive relation between substantial obesity and low levels of depression in males but not in females [25]. Additionally, other studies in China have found that the elderly population with a normal weight is more likely to suffer from depressive symptoms than the obese elderly population [17, 23].

A substantial volume of literature has reported an association between obesity and depression. Some studies have reported no association between obesity and depression [26, 27], while others have demonstrated that obesity was associated with an increased risk of depression; moreover, some studies have found a positive association in only females [28, 29], and some found have that the association is stronger in females than in males [13, 30].

The “jolly fat” hypothesis can be applied to explain the inverse association between obesity and depressive symptom onset [25]. One possible explanation is that food deprivation may lead to depression in people seeking to lose weight through the restriction of diet, although this may occasionally be a protective mechanism against the experience and display of anxiety and depression among people who are obese and experience periodic overeating. In addition, overweight males have been shown to be more content than other groups [25].

There is evidence that males are prone to judge a smaller body frame as less preferable than a larger, more muscular body frame. In addition, compared to a heavier body weight, more males regard low body weight as a poor body image. To this end, it is undesirable for men to have progressively lower relative body weights, a view that may be reinforced by societal standards. In fact, males who are in the overweight or obesity category feel more satisfied with their body because of the belief that a large and muscular body size corresponds to the current norms of masculinity [31].

Previous studies have demonstrated a dose-response relationship between obesity and adverse physical health outcomes. Therefore, there may exist a nonlinear relationship between the psychological risks of obesity and obesity. People with obesity are more likely to die at younger ages; thus, due to the survival effect, obese people living through their middle age may have survival genes protecting them from depression [14].

Regarding Chinese people, Chinese traditional culture may also provide other plausible explanations for the “jolly fat” hypothesis. Chinese people are more likely to have positive perceptions of obesity because it is considered good fortune to become fat during middle age in traditional Chinese culture [32].

Meanwhile, the prevalence of central obesity (73.65% in females and 49.35% in males) in our study differed from that reported in other studies from China. One possible reason for this difference is that the other studies used a more stringent criteria of WC (≥90 cm in males and ≥ 85 cm in females) [33]. Another reason is that the study sample did not exhibit depressive symptoms at baseline, and as we have found that there is a negative association between obesity and depression, it is reasonable that the prevalence of obesity was higher among our cohort than among that of other studies.

BMI has been proven to be a cheap, convenient and common tool to screen overweight and obesity. However, BMI measures are limited because they do not discriminate between fat and lean mass. Thus, the prevalence of obesity defined by excess body fat, particularly in overweight individuals, may be underestimated using BMI [34]. Both general and abdominal obesity assessments are important and necessary to improve the evaluation of potential health risks and to provide an accurate prognosis by estimating fat distribution. It is reported that people at risk tend to store their excess weight abdominally; thus, measurements of WC may help improve their treatment [35]. In this study, we used both general obesity (BMI) and abdominal obesity (WC) to examine the relationship between obesity and depression, and we found that the WC showed equivalent results to BMI. We also observed a significant relationship between obesity and depression in males but not in females. Therefore, in assessing patients with depression risk associated with obesity, measuring WC could be an alternative choice to measuring BMI.

Even though the association between obesity and depression in females was not statistically significant, this finding is consistent with that of previous epidemiological studies focusing on elderly people, as mentioned above. Nevertheless, we found that the prevalence of obesity and depression among females was higher than that among males, which is also consistent with the published literature [36, 37]. Owing to dietary patterns, females have a higher risk of obesity/central obesity than males [38]. In addition, females are more likely to be depressed than males because of hormonal fluctuations, such as undue sensitivity and hormonal changes during menopause [17].

Several limitations of this study should be noted. First, the CES-D-10 was used to examine the number of self-reported depressive symptoms during the past week, which could have introduced recall bias. However, the CES-D-10 is a commonly used tool to evaluate clinically significant depressive symptoms. Second, the severity of depression could not be calculated, which may have introduced bias, as the association between obesity and depression may be stronger among participants who are more depressed. Third, this article adopted the Chinese criteria of obesity status; as a result, the results of this research cannot be generalized to other ethnic groups.

## Conclusion

In conclusion, obesity is more likely to be associated with the onset of depression in males than in females. Additionally, measuring WC could be an alternative to measuring BMI when examining the association between obesity and depression. The impact of the association between obesity status and depression seems to be of sufficient importance to encourage health care providers to monitor mood status among people who are seen as needing weight loss; thus, providers should take into consideration mental changes and the rapid loss of weight.

## Abbreviation

HS: Hukou status
